# Exploring microbiome diversity between behavioural strategies in a facultatively parasitic mite

**DOI:** 10.1017/S0031182026102121

**Published:** 2026-05

**Authors:** Emily Shea Durkin, Anngelyk M. La Luz Maldonado, Carl Nick Keiser

**Affiliations:** 1 Department of Biology, University of Tampa, Tampa, FL, USA; 2 Department of Biology, University of Florida, Gainesville, FL, USA

**Keywords:** Acari, alternative strategies, microbial community, parasitism, phoresy

## Abstract

Parasitic arthropods often depend on symbiotic microbes to supplement their narrow diets. Facultative parasites exhibit variation in their parasitic activity and diet, and thus, might be expected to have greater variation in their microbial communities. Further, individuals that engage in more parasitic activity may have different microbial communities from those less parasitic within the same population, but this remains unexplored. Here, we compared the microbial communities of individuals exhibiting parasitic (*n* = 30) and nonparasitic (*n* = 29) tendencies from two populations (one originating from Tampa, FL and the other Gainesville, FL) of facultatively ectoparasitic mites (*Macrocheles muscaedomesticae*). Microbial alpha diversity was similar across mites, regardless of parasitic activity or population. Using ANOSIM, we found that our dataset clustered into four groups. The composition of microbial communities of non-parasitic *M. muscaedomesticae* mites originating from Tampa and Gainesville was distinct from each other, whereas the parasitic mites had a much greater degree of overlap. We hypothesize that the association of parasitic individuals with fly hosts drove the observed overlap in their microbial communities.

## Graphical abstract

**Figure:**
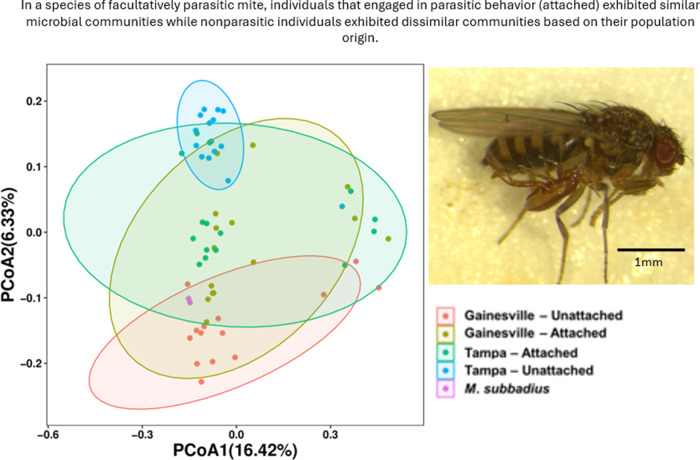

## Introduction

Microbes play an important role in the biology of arthropods by providing their hosts with nutrients (Husnik, 2018; Lee et al., 2020), influencing their behaviour (Lewis and Lizé, 2015; Hosokawa and Fukatsu, 2020), and impacting their reproductive biology (Engelstädter and Hurst, 2009). Conversely, an individual’s physiology, diet, or environment can impact their microbiome (Yun et al., 2014; Mason, 2020; Lange et al., 2023). Ectoparasitic arthropods often have narrow diets and thus rely on microbial symbionts to provide them with necessary nutrients (Sonenshine and Stewart, 2021). For example, vertebrate blood-feeding arthropods receive necessary B vitamins not from their bloodmeals but rather from bacterial symbionts (Husnik, 2018; Speer et al., 2020). Host blood is a major source of microbes colonizing the guts of hematophagous arthropods (Swei and Kwan, 2017; Muturi et al., 2021). Studies examining the microbiomes of individual ectoparasites have revealed that host identity explains a large proportion of microbiome variation between individuals (Swei and Kwan, 2017; Landesman et al., 2019; Doña et al., 2021; Muturi et al., 2021). On the other hand, some ectoparasites maintain a core microbiome with no influence of host identity (McCabe et al., 2020). To date, analyses on the microbial communities of parasitic arthropods have focused on obligate parasites only. To our knowledge, the microbial communities of facultatively parasitic arthropods have not yet been explored, and the variation in their microbial communities could contribute to the variation in their parasitic strategies (Poulin et al., 2023).

Facultative parasites are organisms that can engage in a parasitic relationship with a host organism, but they do not rely on that parasitic relationship for survival or reproduction. Obligate parasites have an intimate/obligate relationship with their host, and evidence suggests their microbial community is shaped by this consistent relationship (Speer, 2022: Xuhong et al., 2025). Facultative parasites, on the other hand, exhibit a range in their level of intimacy with their hosts. Some individuals may never interact with a host in their lifetime, and others may interact with them regularly. The role of these different behavioural strategies on arthropod microbial communities remains unexplored. It could be that individuals that engage in parasitic behaviour have more diverse microbial communities because they utilize and encounter multiple resources and environments (host tissues and environmental resources) compared to individuals that remain solely free-living. For example, Florkowski and Yorzinski (2023) observed in a population of house sparrows that individuals that engaged in more exploratory behaviour exhibited the greatest microbial diversity (Florkowski and Yorzinski, 2023). Alternatively, a core microbiome may be maintained regardless of behavioural strategy.

The facultatively parasitic mite, *Macrocheles muscaedomesticae* (Scopoli 1772), is commonly found in decomposing habitats where it consumes small invertebrates (Jalil and Rodriguez, 1970). When offered the opportunity, female mites may attach to larger, more mobile arthropods, primarily as a means for dispersal (phoresy), and there is evidence to suggest that some mites may obtain a host meal while attached (Jalil and Rodriguez, 1970; Abo-Taka et al., 2014; Durkin et al., 2019). However, prior research shows that *M. muscaedomesticae* may not be generalists but rather show individual variation in parasitic attachment propensity that is partially due to genetic underpinnings (Durkin and Luong, 2018; Durkin et al., 2020). In other words, some individual mites consistently attach to hosts while others never attach to a host. Mites exhibiting different host attachment behaviours may exhibit differences in their microbial communities. Here, we sequenced the microbial communities of facultatively parasitic mites exhibiting parasitic and non-parasitic behaviours collected from two populations. Our objective was to compare the abundance and diversity of microbes between individual mites engaging in parasitic behaviour vs free-living behaviour, and compare the influence of behavioural strategy vs population origin.

## Methods

### Animal collection and maintenance

We maintained two population cultures of *M. muscaedomesticae* generated from wild-collected mites in Tampa and Gainesville, FL, in Summer and Fall 2022. Mites were collected by sweep-netting fruit flies on compost piles and collecting attached mites. These source populations are approximately 250 km apart but with similar habitat types. The Tampa *M. muscaedomesticae* laboratory population was initiated with 30 female mites from a single residential compost collection site (27.719601°N, −82.451879°W) in July 2022. The Gainesville laboratory population was initiated with 25 female mites from a single residential compost collection site (29.623039°N, −82.336848°W) in November 2022. All mite populations were maintained in a single, separate ventilated 4 L plastic containers with wheat bran and wood chips moistened with distilled water and inoculated with unidentified wild-caught nematodes isolated from Tampa compost (27.719601°N, −82.451879°W) using a Baerman funnel (Tintori et al., 2022) for food (Durkin and Luong, 2018). Fresh ingredients were added weekly. For the duration of the behavioural assays both mite cultures were maintained at ambient lab conditions in Tampa, FL. Host fly (*Drosophila hydei*; Sturtevant 1921) cultures were ordered from Josh’s Frogs and maintained on their *D. hydei* fruit fly media (https://joshsfrogs.com/sp/josh-s-frogs-hydei-fruit-fly-media-3-lbs-2-7-quarts-makes-20-fruit-fly-cultures-jf00502). We also maintained a population of *Macrocheles subbadius* (Berlese 1904) mites started with 17 females collected from a single residential compost pile in Tampa, FL (27.921343°N, −82.497941°W) in April 2022. *M. subbadius* mites were differentiated from *M. muscaedomesticae* morphologically by slide mounting individuals in polyvinyl alcohol and identified using Halliday (1990). *M. subbadius* mites did not go through the behavioural assay process, but a small number of samples were processed the same as other mites before DNA extraction as a species comparison and outgroup.

### Host attachment assays

Adult female *M. muscaedomesticae* mites were haphazardly selected from cultures for behavioural analysis. We tested the attachment behaviour of each mite three times, 24 h apart. After each behavioural test, mites were individually placed in a labelled 29.5 mL plastic deli cup filled with 10 mL of nematode culture medium (Durkin et al., 2020).

Behavioural assays were performed at ambient lab conditions (22–24°C, 45–60% RH) between 09:00 and 16:00 h. Each mite was placed into an exposure chamber, constructed from a 200 uL pipette tip reduced to half its length (∼1.5 cm) stoppered with cotton with a single *D. hydei* fly. Host fly age and sex was not controlled. To reduce heterogeneity in host resistance behaviour, the cotton was pushed far enough into the chamber to limit fly mobility while still allowing the mites free movement. Mites were exposed to an individual *D. hydei* fly for 60 min and then scored as ‘attached’ or ‘unattached’ to their fly host at the conclusion of the assay. We refer to these as ‘behavioural strategies’ as prior evidence suggests that individuals are consistent in their attachment propensity (Durkin et al., 2020) and that attachment behaviour has a genetic component (Durkin and Luong, 2018). Fly and mite pairs were then anesthetized using CO_2_ in their exposure chambers and returned to their labelled cup with nematode media using a small paintbrush. If the mite was attached to the fly, the mite was brushed off the fly while anesthetized. Thus, mites were allowed 60 min maximum to remain attached to the fly during the behavioural assay periods. From the Tampa, FL population, 56 mites attached at least once during the 3 exposures and 28 mites never attached during the three exposures. From the Gainesville, FL population, 21 mites attached at least once, and 52 mites never attached.

After the third and final round of host exposure, the fly/mite pair was anesthetized using CO_2_ and the mite only was placed in a container using a small paintbrush for a ‘starvation period’. In previous investigations, the microbial communities of these mites were dominated by environmental bacteria present in the nematode culture medium (unpublished data) so we maintained the mites outside the culture medium for 48 h (starvation period) before molecular processing. A starvation container consisted of a labelled 29.5 mL plastic cup containing moistened plaster. All mites used for molecular analyses experienced a starvation period of 48 h. If a mite died during the starvation period, it was discarded and not used for molecular analysis. Mites were maintained at ambient lab conditions (22–24 °C, 45–60% RH) under natural light cycles for the starvation period. After the starvation period, live mites were individually placed in labelled 1.5 mL microcentrifuge tubes containing 70% EtOH.

### DNA extraction and 16s sequencing

DNA extraction was performed in July 2023. Thus, the Tampa *M. muscaedomesticae* population was in culture 12 months, the Gainesville *M. muscaedomesticae* population 9 months, and the *M. subbadius* population 15 months when molecular work began. Prior to DNA extraction, each mite was surface sterilized. The 70% EtOH the mite was stored in was poured off and the 1.5 mL microcentrifuge tube was filled with distilled H_2_O and shaken for 10 sec. Using a plastic transfer pipette, the distilled H_2_O was removed from the tube, filled with 0.5% bleach solution, and shaken for 10 sec. Using a plastic transfer pipette, the bleach solution was removed from the tube, filled with distilled H_2_O and shaken for 20 sec. The efficacy of similar techniques has shown to be effective in removing external contaminants (Lacey, 1997; Huszarik et al., 2023; Jüds et al., 2023). Immediately after washing, individual mite DNA was extracted using the ZYMO Quick-DNA Fungal/Bacterial miniprep kit. Mite DNA samples were stored at 4 ℃ prior to sequencing.

From both populations, we extracted bacterial DNA from 20 mites that attached at least once and 20 mites that never attached to a fly host. DNA was sent to LCSciences (Houston, TX) for microbial sequencing and bioinformatics analyses. Universal bacterial primers (341 F/805 R) which target the V3 and V4 regions of 16S rDNA were used which generated a 465 bp amplicon. The amplified library was sequenced on a NovaSeq platform with 250 bp paired-end reads. Raw FASTQ files were subject to reads merge by overlapping sequences, data quality control, and chimera filtering by LCSciences, and DADA2 was used for dereplication and generation of representative sequences at single-base resolution (Callahan et al., 2016).

### Statistical analyses

Microbiome data were analysed by LCSciences using the QIIME 2 platform (Bolyen et al., 2019). Comparisons were made between *M. muscaedomesticae* populations and mite behavioural types, and we excluded *M. subadius* from formal comparisons due to small sample size. To test whether bacterial alpha diversity indices differed between mite populations and behavioural strategies, we analysed bacterial amplicon sequence variant (ASV) richness, Shannon diversity index, and Simpson’s diversity index with three separate general linear models with the following independent variables: mite population ID, mite behavioural strategy, and a population x behaviour interaction term. We chose to compare samples using three alpha diversity indices because they each assess different aspects of community diversity (e.g. evenness and dominance; Kers and Saccenti, 2022). GLMs were performed in JMP Student Edition. To test differences in bacterial community composition between populations and behavioural strategies, we used ANOSIM with 999 permutations, which tests whether between-group variation is significantly greater than within-group variation. Unweighted unifrac distances between samples were calculated in QIIME2 and visualized via PCoA. Lastly, we tested for clustering in microbial communities between mite populations and behavioural strategies using SILVA and NT-16S database for taxonomy with a confidence level > 0.7. Clustering at the phylum level was based on Bray-Curtis distances.

## Results

From the Tampa population, sequencing was successful for 30/40 submitted mites (15 attached and 15 non-attached). From the Gainesville population, sequencing was successful for 29/40 submitted mites (15 attached and 14 non-attached). Five *M. subbadius* mites were submitted for sequencing, but only two were successful. We identified 438 bacterial Amplicon Sequence Variants (ASV) unique to attaching mites and 397 ASVs unique to unattached mites, with 242 ASVs shared between the groups. Estimates of microbial alpha diversity (Richness, Shannon’s and Simpson’s) of mite microbial communities did not differ significantly between attaching and non-attaching *M. muscaedomesticae* mites, nor did they differ between the two mite populations (all *p* > 0.34; Table 1). In other words, microbiome diversity was similar across mites, regardless of attachment behaviour or population. Although we cannot compare the *M. muscaedomesticae* and the *M. sabbadius* mites statistically with only two samples for the latter, we can report that ASV richness in the *M. sabbadius* samples was over three times greater than that of *M. muscaedomesticae.*

**Table 1.** Alpha diversity indices. Results of general linear models predicting three estimates of microbial alpha diversity: OTU richness, Shannon diversity and Simpson’s index. All DF = 1.58 Table 1 long description.

Variable	*F*-value	*p*-Value
*OTU Richness*		
Population	0.52	0.47
Behaviour	0.58	0.45
Population × behaviour	0.11	0.74
*Shannon diversity*		
Population	0.66	0.42
Behaviour	0.13	0.72
Population × behaviour	0.84	0.36
*Simpson’s index*		
Population	0.92	0.34
Behaviour	0.02	0.9
Population × behaviour	0.75	0.39

**Figure 1.**
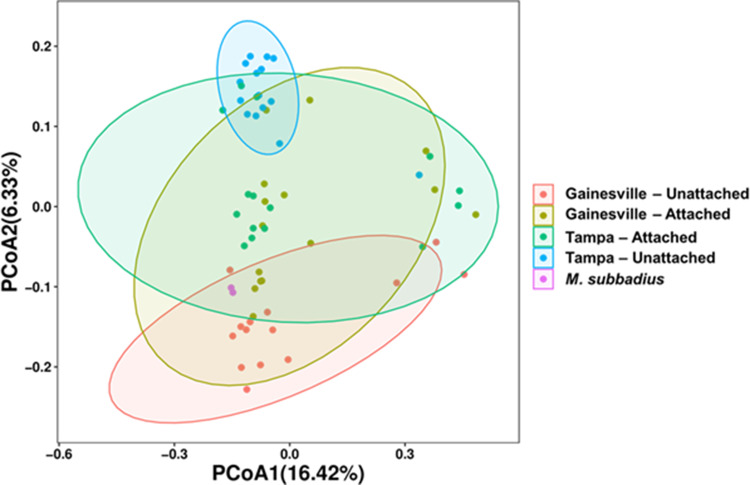
Principal coordinate analysis plot showing four clusters of *Macrocheles muscaedomesticae* mite microbial communities based on two behavioural strategies from two different populations. Attaching mites from two different populations had overlapping microbial communities whereas unattached mite communities were distinct. The two purple points represent samples from a congener mite, *M. subbadius*. Figure 1 long description.

Using ANOSIM, we found that our dataset clustered into four groups (*R* = 0.25, *p* = 0.001; Fig. 1). PCoA axis 1 explained 16.42% of the total variation in microbial community composition, and PCoA2 explained a further 6.33%. We found that the microbial communities of unattached mites from different populations were distinct from each other, whereas the attaching mites from both populations had a much greater degree of overlap in their microbiome composition.

**Figure 2.**
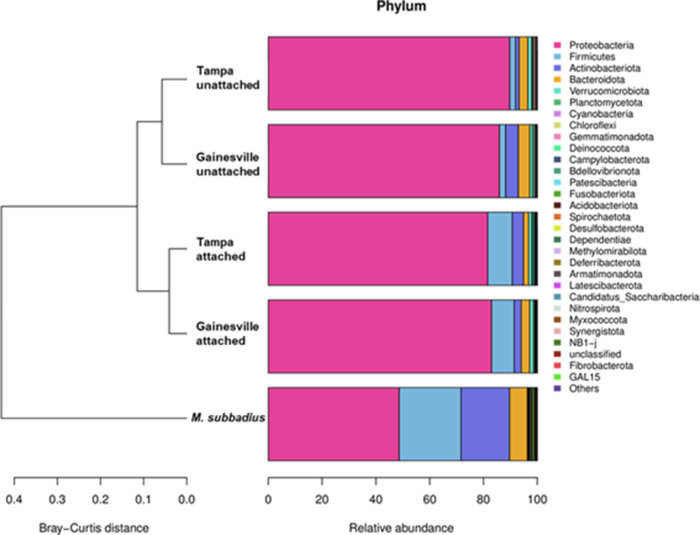
Clustering based on Bray-Curtis distances shows that unattached Macrocheles muscaedomesticae mite microbial communities clustered together separately from attached mites from both populations, while the M. subbadius samples represented an outgroup. Figure 2 long description.

At the phylum level, all samples were dominated by Proteobacteria, but clustering based on Bray−Curtis distances demonstrated that unattached mites clustered together separately from attached mites from both populations, while the *M. subbadius* samples represented an outgroup (Fig. 2).

## Discussion

The composition and function of parasite microbiomes are an area of rapid development in parasitology (Dheilly et al., 2017). As far as we are aware, all previous studies on parasite microbial communities have focused on obligate parasites, especially endoparasites. Here, we explored the microbial communities associated with a facultatively ectoparasitic mite from two different populations and behavioural strategies.

We found no significant difference in microbial alpha diversity between mites exhibiting different parasitic behavioural strategies or in either population. In other words, attaching and non-attaching mites had similar microbial communities in terms of ASV richness across both populations. Studies have attributed differences in microbial communities between individual arthropods to differences in diet or habitat (Kolasa et al., 2019; Lange et al., 2023). The individuals in our study were mass-reared in the same lab space on the same food source, reducing the heterogeneity in diet and environment and likely reducing differences in alpha diversity.

When examining beta diversity between microbial communities, mites exhibiting parasitic behavioural strategies had more similar microbial community composition regardless of their population origin. The non-attaching mites from the two populations did not overlap, indicating some inherent differences in microbial community composition due to their population origin. This is particularly interesting given that these mites were maintained in similar laboratory conditions for several generations prior to this experiment. Small et al. (2023) found that the source population explained a large portion of microbiome variation in threespine sticklebacks after maintenance in a common garden, which they attributed to genetic background, and ground beetle gut microbiomes are similarly resilient to change after laboratory diet manipulation (Silver et al., 2021). However, we must acknowledge that microbial drift, founder effects and/or container-specific differences could have driven their microbial differences. Attaching mites, on the other hand, had overlapping microbial communities despite their differing population identity. There are at least two potential explanations for this result. First, attached mites consumed a common food resource: fly tissue/haemolymph (Polak, 1996). It is well recognized that an organism’s diet can have a significant impact on its microbial community, including predatory mites (Yan et al., 2021; Lange et al., 2023). Fly tissue could serve as a source for novel microbes, thereby shifting the existing microbial community. Currently, there is no definitive evidence for this species of mite ingesting fly tissues while attached, but there are studies with data that suggest it is likely (Jalil and Rodriguez, 1970; Polak, 1996; Abo-Taka et al., 2014; Durkin et al., 2019). Even if consumption of fly tissue is not a driving factor in microbiome similarity, their chelicerae are used for host attachment and could become exposed to fly cuticular microbes. Characterizing the microbial communities of attaching mites and their fly hosts and recording the length of time a mite was attached could bring more insight into this hypothesis. Alternatively, microbial community composition may influence the likelihood of mite attachment to flies. Ectoparasite microbiomes may alter feeding preferences (Speer, 2022), and experimental inoculations with gut microbes may test this hypothesis.

The microbial communities of *M. muscaedomesticae* mites were dominated by members of the Phylum Proteobacteria (Fig. 2). This is not surprising, as Proteobacteria commonly dominate mite microbial communities (Chaisiri et al., 2015; Yan et al., 2021) and likely arise from the laboratory culture diet and substrate. Mites were starved outside of culture substrate for 48 h and then surface sterilized with a series of water and bleach solution rinses before DNA extraction (Huszarik et al., 2023; Jüds et al., 2023). However, we cannot exclude the possibility of environmental microbes influencing the makeup of their microbial communities. Following in relative abundance were bacteria from the Firmicutes, Actinobacteria and Bacteroidota. We only sequenced two individuals of *M. subbadius* and, therefore, must be cautious in our interpretation, but the relative abundance of Proteobacteria was much smaller in these mites. The *Macrocheles* mites provide an interesting system in which to test the role of lifestyle plasticity (parasitic vs free living) on host-microbe cophylogenetics.

Overall, we found evidence for differing microbial communities between individuals exhibiting parasitic and non-parasitic behaviours in facultatively parasitic mites. The mites with parasitic tendencies showed similarities in their associated microbial communities despite having been collected from two different source populations, while the microbial communities of non-attaching mites differed between populations. We hypothesize that association with fly hosts drove the similarities in the microbial communities of parasitic mites. As *M. muscaedomesticae* have been found to vector bacteria between fly hosts (Stone et al., 2024), the data presented here add an important perspective on the natural history of facultatively parasitic mites and their role in host–pathogen interactions.

## Table 1 Long description

The table presents results from general linear models predicting microbial alpha diversity using three indices: OTU richness, Shannon diversity, and Simpson's Index. For OTU richness, population and behavior have similar F values of 0.52 and 0.58, respectively, with non-significant p-values. Shannon diversity shows population with an F value of 0.66, while behavior is lower at 0.13, both with non-significant p-values. Simpson's Index has the highest F value for population at 0.92, but again, p-values indicate no significant effects. Across all indices, interactions between population and behavior show low F values and high p-values, suggesting no significant interaction effects. Overall, the data suggests that neither population nor behavior significantly influences microbial alpha diversity in this study.

Navigate back to Table 1.

## Figure 1 Long description

The scatter plot illustrates microbial community composition using Principal Coordinates Analysis. The x-axis is labeled PCoA1 with 16.42 percent and the y-axis is labeled PCoA2 with 6.33 percent. Different colored ellipses represent distinct clusters: Gainesville Unattached, Gainesville Attached, Tampa Attached, Tampa Unattached and M subbadius. Each cluster is marked with specific symbols and colors, showing the distribution of data points within the plot.

Navigate back to Figure 1.

## Figure 2 Long description

The image shows a bar graph with six horizontal bars representing the relative abundance of various phyla in different mite samples. The samples are labeled as Tampa unattached, Gainesville unattached, Tampa attached, Gainesville attached and M. subbadius. Each bar is divided into segments representing different phyla, with Proteobacteria being the most dominant. A dendrogram on the left illustrates the clustering of samples based on Bray-Curtis distances, showing Tampa unattached and Gainesville unattached clustering together, Tampa attached and Gainesville attached clustering separately and M. subbadius as an outgroup. The x-axis of the bar graph is labeled 'Relative abundance' ranging from 0 to 100, while the x-axis of the dendrogram is labeled 'Bray-Curtis distance' ranging from 0.0 to 0.4.

Navigate back to Figure 2.
